# Deciphering bacterial and fungal endophyte communities in leaves of two maple trees with green islands

**DOI:** 10.1038/s41598-019-50540-2

**Published:** 2019-10-02

**Authors:** Franziska Wemheuer, Bernd Wemheuer, Rolf Daniel, Stefan Vidal

**Affiliations:** 1 Department of Crop Sciences, University of Göttingen, Grisebachstr.6, D-37077 Göttingen, Germany; 2 Applied Marine and Estuarine Ecology, Evolution and Ecology Research Centre, School of Biological, Earth and Environmental Sciences, University of New South Wales, Sydney, Australia; 3 Genomic and Applied Microbiology and Göttingen Genomics Laboratory, Institute of Microbiology and Genetics, University of Göttingen, Grisebachstr. 8, D-37077 Göttingen, Germany; 4 Centre for Marine Science and Innovation and School of Biological, Earth and Environmental Sciences, University of New South Wales, Sydney, Australia

**Keywords:** Metagenomics, Fungal ecology, Microbial ecology

## Abstract

Green islands (the re-greening of senescent leaf tissues) are particularly evident on leaves infected with fungal pathogens. To date, there is only a limited number of studies investigating foliar endophytic microorganisms in phytopathogen-infected leaves. Here, we analysed bacterial and fungal endophyte communities in leaves without green islands (control leaves; CL), within green island areas (GLA) and the surrounding yellow leaf areas (YLA) of leaves with green islands of *Acer campestre* and *A. platanoides*. GLA samples of *A. campestre* and *A. platanoides* were dominated by *Sawadaea polyfida* and *S. bicornis*, respectively, suggesting that these fungi might be responsible for the green islands. We detected a higher fungal richness and diversity in CL compared to GLA samples of *A. campestre*. Leaf status (CL, GLA, YLA) significantly altered the composition of fungal communities of *A. campestre*. This was related to differences in fungal community composition between YLA and GLA samples. Site was the main driver of bacterial communities, suggesting that bacterial and fungal endophytes are shaped by different factors. Overall, we observed *Acer* species-specific responses of endophyte communities towards the presence of green islands and/or leaf type, which might be attributed to several fungi and bacteria specifically associated with one *Acer* species.

## Introduction

Endophytic microorganisms, including bacteria and fungi, have been detected in all plant species investigated so far^1^. Over the past years, the definition for endophytes has been controversially used in literature. According to Le Cocq *et al*.^2^ and van Overbeek and Saikkonen^3^, endophytes are those ‘microbes which colonize internal plant tissues for at least parts of their life cycle without causing disease symptoms under any known circumstances’. In contrast, Hardoim *et al*.^1^ suggested that the term “endophyte” should refer to their habitat only but not to their function. Here, we adopt the concept of Hardoim *et al*.^1^, which includes both pathogenic and non-pathogenic endophytes.

Previous investigations on endophyte communities using high-throughput sequencing-based approaches have led to novel insights into phylogenetic and functional responses of these communities to a diverse array of factors, such as plant species and/or plant compartment^4–7^. Moreover, these techniques significantly contributed to our understanding of plant-microbe interactions^4,7,8^ and microbial interactions in plants^5,8,9^. These interactions can involve not only plant-beneficial microorganisms but also phytopathogens^10^.

A phenomenon, typically referred to as “green islands” of plant tissues, is frequently observed in plant-pathogen interactions^11–13^, i.e., the sites of pathogen infection remain green whilst the surrounding leaf tissues senesces^13^. Green islands are particularly evident on leaves infected with obligately biotrophic as well as necrotrophic fungal pathogens, such as rusts or powdery mildew^11,12,14^. It has been assumed that green islands are beneficial to the fungal pathogen by prolonging the life of host cells and, as a consequence, its period of reproduction^13^. Previous studies reported that cytokinins promote re-greening of senescent leaf tissues^15,16^. Interestingly, leaf mining insects can also induce green islands^17,18^. However, a previous study on green islands and leaf-mining insects suggested that the insect bacterial symbiont *Wolbachia* plays a key role in green-island induction by manipulating the cytokinin levels of plants^19^.

According to Tian *et al*.^20^, studies on bacterial endophytes in healthy plants are not sufficient to understand the dynamics of plant-microbiome-pathogen relationships in the absence of plant pathogens. So far, only few studies have investigated endophyte communities in diseased plant tissues^21–24^. Recently, de Assis Costa *et al*.^25^ analysed the fungal diversity of asymptomatic and symptomatic leaf samples of the oil palm *Elaeis guineensis*. Here, the fungal community composition in asymptomatic leaves was more similar to each other compared to symptomatic leaves. In another study by Bogas *et al*.^21^, a higher bacterial endophyte diversity was observed in *Colletotrichum* spp. infected *Paullinia cupana* trees. The authors suggested that there is a possible interaction between anthracnose and the endophytic bacterial community. Deveau *et al*.^26^ proposed that interactions between bacteria and fungi contribute to both diseases and health of plants. However, our knowledge on bacterial-fungal interactions in diseased plant tissues remains incomplete, as most previous studies have not simultaneously analysed fungal or bacterial endophyte communities^9,22–24,27^.

In the present study, we investigated fungal and bacterial endophytes and their interactions in leaves with and without green islands of field maple (*Acer campestre* L.) and the temperate Norway maple (*A. platanoides* L.) using large-scale metabarcoding. For this purpose, leaves of *A. campestre* and *A. platanoides* trees were collected at three sampling sites near Göttingen, Lower Saxony, Germany. Both *Acer* species were chosen because they are broadly distributed in Europe and the green areas on the leaves become very conspicuous in autumn, when the leaves turn to bright yellow. Bacterial and fungal endophyte communities in control leaves (CL) without green islands as well as within green leaf areas (GLA) and in the surrounding yellow leaf areas (YLA) of leaves with green islands were examined using Illumina (MiSeq) sequencing targeting the bacterial 16S rRNA gene and the fungal internal transcribed spacer region 2 (ITS2), respectively. To better understand plant-microbe interactions in leaves with and without green islands of both *Acer* species, correlation-based indicator analysis^28^ was performed.

Our major aims were as follows: (i) to examine the assemblage of foliar endophyte bacteria and fungi of *A. campestre* and *A. platanoides*, (ii) to assess the effect of green island presence on microbial diversity and community structures, (iii) to examine whether this effect differs between *A. campestre* and *A. platanoides* and (iv) to determine if fungi and bacteria are affected in the same ways by the presence of green islands. To our knowledge, this is the first report on bacterial and fungal endophyte communities in leaves without and with green islands.

## Results

**Figure 1:**
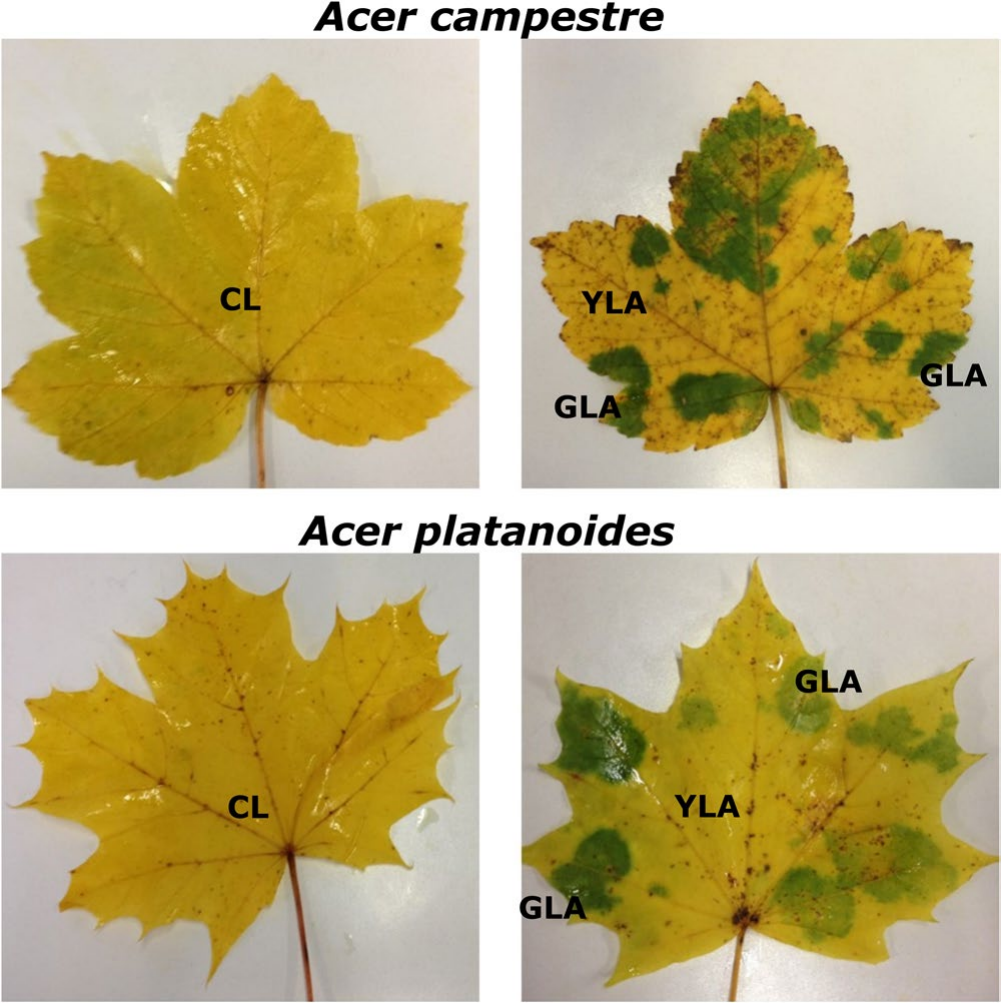
Leaves of *A. campestre* and *A. platanoides* without (control leaves; CL) and with green islands. Leaves with green islands contained green (GLA) and yellow leaf area (YLA) samples.

Leaves with and without green islands (Fig. 1) of *A. platanoides* and *A. campestre* trees were collected at three sites near Göttingen, resulting in a total of 65 leaf samples (Table 1, Supplementary Material Table S1). After removal of low-quality reads, PCR artefacts (chimeras), singletons and plant-derived contaminations, a total of 1,437,845 and 4,337,507 high-quality reads were obtained for bacteria and fungi, respectively (Supplementary Material Tables S2, S3). Sequence numbers per sample varied between 8,061 to 62,942 (average 22,121) for bacteria and 11,932 to 164,642 (average 66, 731) for fungi, respectively (Supplementary Material Tables S4, S5).

**Table 1:** Study design and sample numbers investigated in this study. Leaves of ***A. platanoides*** and ***A. campestre*** with and without green islands were collected at three sites (Molkengrund, Am Kehr and Friedland) in Germany at 1^st^ November 2015.

	Site 1 (Molkengrund)	Site 2 (Am Kehr)	Site 3 (Friedland)	Leaf number	Sample number
***A. platanoides***				**20**	**32**
Control leaves (CL)	4	4		8	8
Leaves with green islands*	6	3		12	24
***A. campestre***				**21**	**33**
Control leaves (CL)		1	8	9	9
Leaves with green islands*	4	3	5	12	24
**Total**	**14**	**11**	**13**	**41**	**65**

*Note that leaves with green islands contained green (GLA) and yellow leaf area (YLA) samples.

Obtained sequences were grouped into 1,176 bacterial and 550 fungal zero-radius operational taxonomic units (zOTUs; denoised, chimera-free sequences with 100% similarity; Supplementary Material Tables S2, S3). Rarefaction curves (Supplementary Material Figs S1a, 2a) and calculated Michaelis-Menten Fit confirmed that the majority of the bacterial (coverage: 86.3%) and the fungal diversity (coverage: 88.4%) were recovered by the surveying effort (Supplementary Material Tables S4, S5). Species accumulation curves further indicated that 92.6% of all fungal zOTUs (maximal number of zOTUs calculated = 594) and 95.3% of all bacterial zOTUs (maximal number of zOTUs calculated = 1,234) were recovered by the surveying effort (Supplementary Material Figs S1b, S2b). This suggests that the library size was large enough to reflect the endophytic bacterial and fungal diversity in leaves of *A. campestre* and *A. platanoides*.

### Bacterial and fungal endophyte communities in leaves are dominated by few phyla

**Figure 2:**
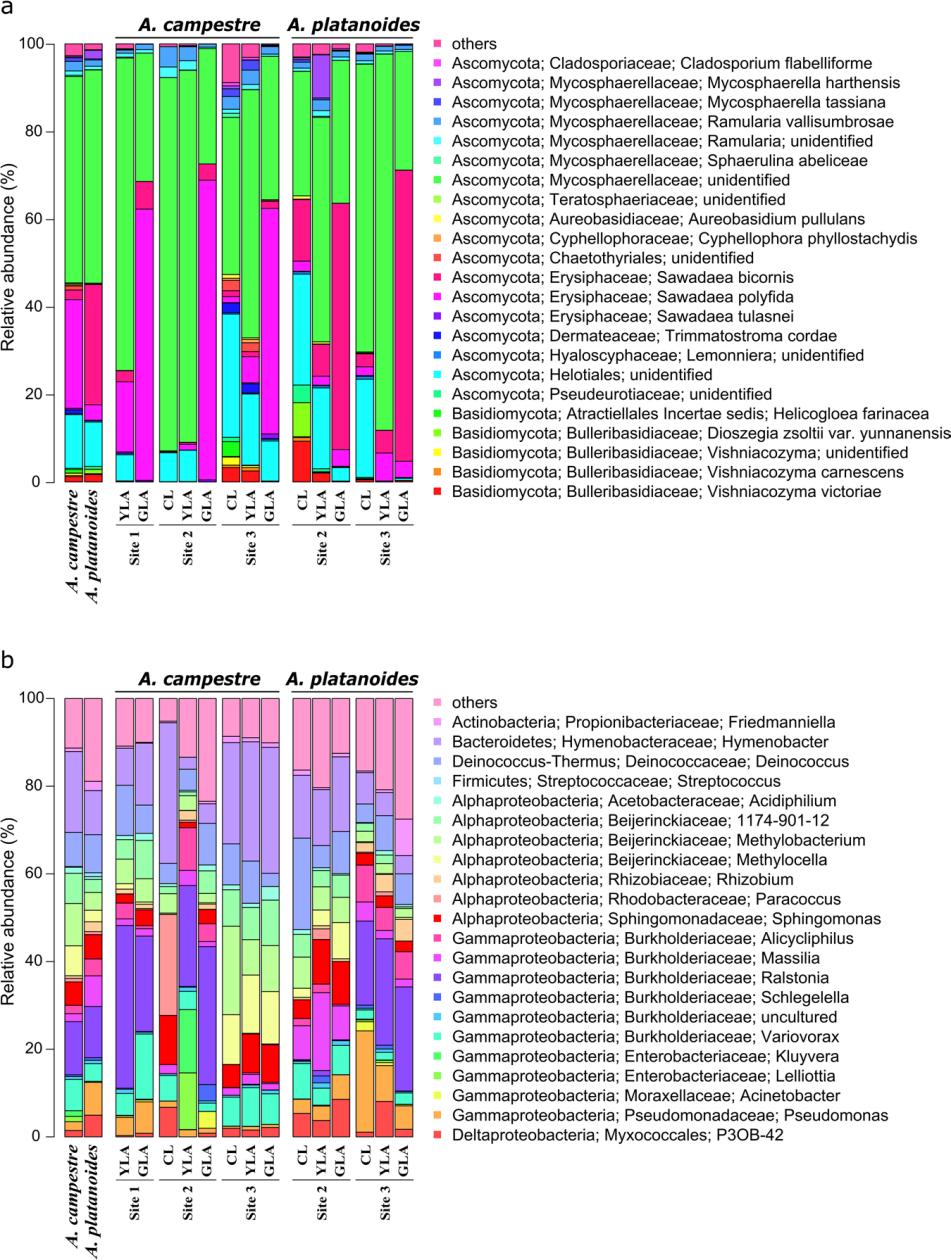
Abundant (**a**) fungal species and (**b**) bacterial genera in leaves without and with green islands of *A. campestre* and *A. platanoides*. Only fungal and bacterial groups with an average abundance of 0.01% and 0.5%, respectively, are shown. Fungal and bacterial groups with an average abundance of less than 0.01% and 0.5%, respectively, are summarized as “others”. Note that *Allorhizobium-Neorhizobium-Pararhizobium-Rhizobium* was shortened for visualization. Abbreviations: CL, control leaves without green islands; YLA, yellow leaf area; GLA, green island area; Site 1, Molkengrund; Site 2, Am Kehr; Site 3, Friedland.

Fungi were represented by two abundant phyla (>0.1% of all sequences across all samples): Ascomycota (mean abundance across all samples: 95.87%) and Basidiomycota (3.11%) (Fig. 2a). The two fungal classes Dothideomycetes (53.41%) and Leotiomycetes (41.60%) dominated the fungal community. At species level, an unidentified member of the Mycosphaerellaceae (47.88%) was predominant. Other abundant fungal species were *S. bicornis* (14.63%), *S. polyfida* (14.34%), one unidentified member of the Helotiales (11.20%), *Ramularia vallisumbrosae* (1.82%), *Vishniacozyma victoriae* (1.44%) and *Mycosphaerella harthensis* (1.08%).

The relative abundances of most fungal species did not differ between both *Acer* species, with exception of *S. bicornis*, *S. polyfida* and *S. tulasnei*. However, several fungal species differed in their abundance with regard to leaf status. Fungi assigned to the family Mycosphaerellaceae were predominant in YLA samples of *A. campestre* (68.59%) and *A. platanoides* (68.56%). In contrast, the fungal species *S. polyfida* (59.18%) and *S. bicornis* (61.32%) dominated the GLA samples of *A. campestre* and *A. platanoides*, respectively. The fungal species *M. harthensis* (4.94%) and *Helicogloea farinacea* (3.01%) were found in YLA of *A. platanoides* and CL of *A. campestre*, respectively, but were detected in very low abundance (<0.1%) in YLA samples of *A. campestre* and in CL of *A. platanoides*, respectively.

Bacterial communities were dominated by Proteobacteria (mean abundance across all samples: 69.34%), Bacteroidetes (14.78%), Deinococcus-Thermus (8.39%), Actinobacteria (4.85%), Firmicutes (2.05%) and Patescibacteria (0.52%) (Fig. 2b). Within the Proteobacteria, Gammaproteobacteria were predominant (37.64%), followed by Alphaproteobacteria (28.13%) and Deltaproteobacteria (3.56%). One unidentified member of the genus *Ralstonia* (10.38%), *Hymenobacter* sp. UV11 (9.10%) and an unidentified member of *Deinococcus* (5.93%) were predominant in the bacterial community. Most bacterial genera, such as *Streptococcus* or *Ralstonia*, did not differ in their relative abundance between both *Acer* species. In contrast, several bacterial genera including *Variovorax*, *Methylocella* or *Methylobacterium* were more abundant in *A. campestre* leaves. The opposite was detected for the genera *Massilia* and *Friedmanniella*. Differences in the relative abundances with respect to *Acer* species and leaf status were also recorded. *Methylobacterium* (18.4%) and *Hymenobacter* (24.11%) were predominant in CL of *A. campestre*, while those of *A. platanoides* were dominated by *Deinococcus* (12.55%) and *Pseudomonas* (13.15%).

### Leaf status significantly affected fungal endophyte diversity in *A. campestre* but not in *A. platanoides* leaves

Changes in alpha diversity values were evaluated by linear mixed effects models with site as random factor and *Acer* species as well as leaf status (model 1) or presence of green islands (model 2). Site significantly affected bacterial alpha diversity values (Supplementary Material Table S6). *Acer* species did not influence bacterial richness and phylogenetic diversity (Table 2, Supplementary Material Table S6), but we detected a significantly higher bacterial diversity in *A. platanoides* than in *A. campestre* (model 1: F_1,54_ = 5.43, P = 0.04; model 2: F_1,55_ = 5.53, P = 0.02). Neither the presence of green islands nor the leaf status influenced bacterial alpha diversity values.

**Table 2:** Alpha diversity measures (mean ± standard deviation) for bacterial and fungal endophytes in leaves with and without green islands of *A. campestre* and *A. platanoides* collected at at three sites (Molkengrund, Am Kehr and Friedland) in Germany. Abbreviations: CL, control leaves without green islands; GIL, leaves with green islands; YLA, yellow leaf area; GLA, green island area; Ac, *Acer campestre*; Ap, *Acer platanoides*.

	Bacterial endophytes			Fungal endophytes	
	Richness	Diversity	Faith’s PD	Richness	Diversity
Ac	244 ± 70	3.15 ± 0.79^A^	5.56 ± 1.31	77 ± 65	1.38 ± 0.83
Ap	242 ± 88	3.38 ± 0.66^B^	5.65 ± 1.71	77 ± 66	1.31 ± 0.77
***Acer campestre***					
Leaves with green islands (GIL)*	229 ± 75	3.03 ± 0.89	5.32 ± 1.44	59 ± 46^A^	1.16 ± 0.66^A^
- Green leaf area (GLA)	228 ± 78	3.07 ± 0.94	5.39 ± 1.48	49 ± 24^a^	1.01 ± 0.45^a^
- Yellow leaf area (YLA)	229 ± 77	2.99 ± 0.87	5.25 ± 1.45	70 ± 61^ab^	1.32 ± 0.81^ab^
Control leaves (CL)	285 ± 25	3.45 ± 0.28	6.22 ± 0.49	123 ± 86^Bb^	1.98 ± 0.98^Bb^
***Acer platanoides***					
Leaves with green islands (GIL)*	238 ± 87	3.35 ± 0.66	5.57 ± 1.67	66 ± 53	1.20 ± 0.67
- Green leaf area (GLA)	230 ± 93	3.25 ± 0.72	5.36 ± 1.64	56 ± 18	1.19 ± 0.31
- Yellow leaf area (YLA)	246 ± 85	3.46 ± 0.61	5.78 ± 1.75	75 ± 73	1.21 ± 0.92
Control leaves (CL)	252 ± 94	3.46 ± 0.71	5.89 ± 1.92	112 ± 92	1.67 ± 0.97
**Am Kehr**					
Ac - CL	280 ± NA	3.15 ± NA	6.09 ± NA	54 ± NA	1.24 ± NA
Ac - GIL	165 ± 57	2.65 ± 1.28	4.21 ± 1.48	43 ± 21	0.99 ± 0.3
Ac - GLA	163 ± 84	2.73 ± 1.72	4.43 ± 2.18	37 ± 7	0.83 ± 0.2
Ac - YLA	168 ± 32	2.57 ± 1.05	3.98 ± 0.78	50 ± 31	1.14 ± 0.35
Ap - CL	209 ± 89	3.15 ± 0.89	4.94 ± 1.59	89 ± 95	1.29 ± 0.99
Ap - GIL	180 ± 61	3.02 ± 0.68	4.62 ± 1.45	40 ± 16	0.94 ± 0.34
Ap - GLA	157 ± 57	2.84 ± 0.73	4.19 ± 1.2	41 ± 10	1.13 ± 0.24
Ap - YLA	203 ± 60	3.2 ± 0.64	5.04 ± 1.67	39 ± 21	0.75 ± 0.34
**Molkengrund**					
Ac - GIL	210 ± 59	2.89 ± 0.96	4.98 ± 1.2	49 ± 35	0.93 ± 0.61
Ac - GLA	217 ± 66	2.94 ± 0.91	5.15 ± 1.42	38 ± 7	0.88 ± 0.65
Ac - YLA	202 ± 60	2.84 ± 1.15	4.81 ± 1.12	60 ± 50	0.98 ± 0.68
Ap - CL	296 ± 89	3.77 ± 0.36	6.84 ± 1.91	135 ± 96	2.04 ± 0.91
Ap - GIL	296 ± 70	3.68 ± 0.46	6.53 ± 1.32	91 ± 65	1.45 ± 0.82
Ap - GLA	304 ± 52	3.66 ± 0.45	6.53 ± 1.11	71 ± 10	1.25 ± 0.38
Ap - YLA	289 ± 88	3.71 ± 0.51	6.53 ± 1.62	111 ± 91	1.66 ± 1.12
**Friedland**					
Ac - CL	286 ± 26	3.49 ± 0.27	6.24 ± 0.52	131 ± 88	2.07 ± 1.01
Ac - GIL	282 ± 62	3.37 ± 0.39	6.26 ± 1.01	77 ± 61	1.45 ± 0.78
Ac - GLA	277 ± 59	3.37 ± 0.36	6.16 ± 0.8	65 ± 30	1.21 ± 0.38
Ac - YLA	287 ± 72	3.36 ± 0.46	6.36 ± 1.28	89 ± 84	1.7 ± 1.03

Alpha diversity values are represented by richness (number of observed zOTUs), diversity (Shannon diversity index H’) and phylogenetic diversity (Faith’s PD). Note that the Faith’s PD was not calculated for fungi due to the high variability in the fungal ITS region.

Abbreviation: NA, not available (standard deviation not available due to the low number of replicates).

*Leaves with green islands (GIL) contained green (GLA) and yellow leaf area (YLA) samples.

^A,B^Different superscript letters indicate significant differences (P ≤ 0.05) between the two *Acer* species (bacterial data) or between leaves with and without green islands (fungal data).

^a,b^Different superscript letters indicate significant differences (P ≤  0.05) between the three leaf states CL, YLA and GLA (fungal data). There was a marginally significant difference in fungal richness between CL and YLA samples of *A. campestre* (P = 0.053) and between CL and YLA samples of the entire dataset (P = 0.054). For further details, see Supplementary Table S6.

In contrast, fungal richness (F_1,62_ = 8.71, P = 0.004) and diversity (F_1,62_ = 7.37, P = 0.01) were significantly higher in control leaves compared to leaves with green islands (Table 2, Supplementary Material Table S6). Similar results were obtained when including the leaf status in the model (richness: F_2,60_ = 5.06, P = 0.01; diversity: F_2,60_ = 3.99, P = 0.02). Multiple comparison using Dunn’s test with *Benjamini-Hochberg* corrected P-values revealed a significantly higher fungal richness (P_adj._ = 0.04) in CL compared to YLA samples. In addition, we observed a significantly lower fungal diversity in GLA (P_adj_ = 0.048) and YLA (P_adj_ = 0.03) than in CL samples. Site and *Acer* species did not influence fungal richness or diversity.

When investigating the endophyte diversity of the two *Acer* species separately, we detected no effect of leaf status or presence of green islands on fungal richness or diversity in leaves of *A. platanoides* and on bacterial alpha diversity measures in leaves of both *Acer* species. However, we detected a significantly lower fungal richness (F_1,28_ = 5.87, P = 0.02) and diversity (F_1,30_ = 5.11, P = 0.03) in green island compared to control leaves of *A. campestre*. The impact of leaf status on fungal richness (F_2,29_ = 3.28, P = 0.052) and diversity (F_2,29_ = 3.11, P = 0.06) of this *Acer* tree species was only marginal. Multiple comparison using *Benjamini-Hochberg* corrected P-values revealed a significantly higher fungal richness (P_adj_ = 0.02) and diversity (P_adj_ = 0.03) in CL compared to GLA samples of *A. campestre*. Moreover, fungal endophyte richness differed marginally (P = 0.053) between CL and YLA samples of this *Acer* tree species.

### The effect of leaf status on fungal community composition differs between the two *Acer* species

As we observed differences in the relative abundances of the predominant fungal species and bacterial genera with regard to *Acer* species and leaf status, we performed non-metric multi-dimensional scaling (nMDS) analyses based on Bray-Curtis and weighted UniFrac dissimilarities for bacteria and Bray-Curtis dissimilarities for fungi (Supplementary Material Fig. S3). We observed that the majority of control leaf samples grouped together in the fungal but not in the bacterial dataset. The effect of site, leaf status and *Acer* species on endophyte community composition was further analysed by permutational multivariate analysis of variance (PERMANOVA). The composition of fungal (Pseudo-F_(2)_ = 3.38, P = 0.001) and bacterial communities (Bray-Curtis: Pseudo-F_2_ = 2.55, P = 0.001; weighted UniFrac: Pseudo-F_(2)_ = 2.9, P = 0.01) was significantly affected by site (Table 3, Supplementary Material Table S7). In contrast, *Acer* species had no significant effect on both bacterial and fungal community composition. Interestingly, green island presence had no effect on the composition of fungal and bacterial endophyte communities. Leaf status affected the composition of the fungal community (Pseudo-F_(2)_ = 5.91, P = 0.04) only. However, multiple comparison based on Bray-Curtis distances revealed only marginally significant differences in fungal community composition between leaf states YLA and GLA (t = 4.11, P = 0.09, permutations = 192).

**Table 3:** Summary of PERMANOVA results for the analysis of differences in endophyte community composition across the different factors (i.e., site, leaf status, *Acer* species and their interactions).

	df*	Bacterial endophyte communities					Fungal endophyte communities				
		SS	MS	Pseudo-F	P -value	perm	SS	MS	Pseudo-F	P -value	perm
**Source**	**Model 1 (entire dataset)**										
*Acer* species (Sp)	1	0.42	0.42	1.06	0.49	30	0.48	0.48	1.01	0.49	30
Leaf status (Le)	2	0.41	0.20	0.81	0.72	993	1.94	0.97	5.91	**0.04** ^**A**^	996
Site (Si)	2	1.45	0.72	2.55	**0.001**	998	1.28	0.64	3.38	**0.001**	999
SpxLe	2	0.57	0.28	1.16	0.47	999	1.31	0.65	2.20	0.26	998
SpxSi	1	0.47	0.47	1.67	0.056	997	0.69	0.69	3.65	**0.005**	998
LexSi	4	1.06	0.27	0.94	0.62	998	0.57	0.14	0.75	0.78	998
SpxLexSi	1	0.23	0.23	0.81	0.69	998	0.34	0.34	1.79	0.10	999
Residual	51	14.46	0.28				9.64	0.19			
Total	64	20.20					20.09				
	**Model 2 (entire dataset)**										
*Acer* species (Sp)	1	0.38	0.38	1.12	0.44	30	0.24	0.24	0.63	0.58	30
Green island presence (In)	1	0.29	0.29	0.77	0.66	333	0.54	0.54	2.16	**0.21**	341
Site (Si)	2	1.11	0.55	2.03	**0.001**	997	0.86	0.43	1.83	0.02	998
SpxIn	1	0.36	0.36	1.30	0.21	999	0.35	0.35	1.47	0.22	998
SpxSi	1	0.47	0.47	1.74	**0.04**	999	0.69	0.69	2.93	**0.01**	998
InxSi	2	0.75	0.37	1.37	0.10	998	0.41	0.21	0.87	0.58	998
Res	56	15.29	0.27				13.16	0.23			
Total	64	20.20					20.09				
	**Model 3 (** ***A. campestre*** **)**										
Leaf status (Le)	2	0.51	0.25	0.93	0.57	972	1.97	0.99	6.83	**0.02** ^**A**^	965
Site (Si)	2	1.01	0.51	1.96	**0.02**	998	1.05	0.52	2.69	**0.004**	999
LexSi	3	0.82	0.27	1.06	0.37	997	0.42	0.14	0.72	0.75	999
Res	25	6.48	0.26				4.86	0.19			
Total	32	9.22					9.41				
	**Model 4 (** ***A. platanoides*** **)**										
Leaf status (Le)	2	0.43	0.22	1.00	0.37	180	2.09	1.04	3.93	0.14	180
Site (Si)	1	1.26	1.26	4.09	**0.002**	998	1.74	1.74	9.47	**0.001**	999
LexSi	2	0.43	0.22	0.71	0.94	996	0.53	0.27	1.44	0.16	996
Res	26	7.99	0.31				4.78	0.18			
Total	31	10.19					9.20				

Data were analysed with site as random effect and *Acer* species as well as leaf status (model 1) or presence of green islands (model 2) as fixed factors. Each *Acer* species was also tested separately with site as random and leaf status as fixed factor (models 3 and 4). Results of the PERMANOVA are based on Bray-Curtis dissimilarities with 999 permutations (perm).

*The degree of freedom (df) is the same for bacteria and fungi.

^A^Multiple comparison revealed a marginally significant difference between all GLA and YLA samples (t = 4.11, P = 0.09, permutations = 192) and a significant difference between GLA and YLA samples of *A. campestre* (t = 4.1, P = 0.048, permutations = 192).

**Figure 3:**
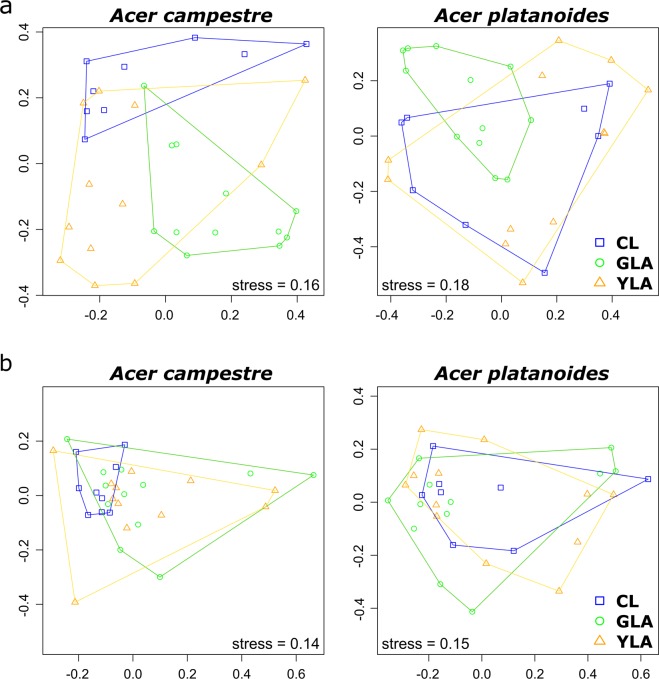
Response of fungal (**a**) and bacterial (**b**) endophyte communities in leaves of *A. campestre* and *A. platanoides* towards green island presence and leaf status. Ordination is based on Bray-Curtis dissimilarities between samples. NMDS ordination of bacterial and fungal communities is color-coded by leaf status. Note that the nMDS axes have different scales for each ordination. NMDS ordination plots for weighted UniFrac dissimilarities can be found in the Supplementary Material as Figure S4. Abbreviations: CL, control leaves; YLA, yellow leaf area; GLA, green island area.

We were also interested in a separate analysis of endophyte community composition in leaves of each *Acer* species. Similar to the results for fungal diversity and richness, we found *Acer* species-specific responses of the fungal community composition towards leaf status (Fig. 3). GLA and CL samples of *A. campestre* grouped together with exception of one sample (Fig. 3a,b). On the other hand, no distinct clustering was observed for bacterial communities of *A. campestre* and *A. platanoides* (Fig. 3c,d) and for fungal communities of *A. platanoides* (Fig. 3a,b). These findings were supported by the results of the statistical analysis (PERMANOVA; Table 3). Leaf status had no effect on the community composition of bacterial endophytes in *A. campestre* and *A. platanoides* and fungal endophytes in *A. platanoides*, but significantly affected those of fungal endophytes in *A. campestre* (Pseudo-F_(2)_ = 6.83, P = 0.02). Multiple comparison based on Bray-Curtis distances revealed significant differences in fungal community composition between leaf states YLA and GLA of *A. campestre* (t = 4.1, P = 0.048, permutations = 192).

Overall, we found different responses of bacterial and fungal endophytes towards site, presence of green islands, leaf status and *Acer* species (Table 4). In addition, the observed effects were not consistent among the *Acer* tree species, as fungal richness, diversity and community structure of *A. campestre* but not of *A. platanoides* differed among the leaf types and/or between leaves with and without green islands.

**Table 4:** Summary of findings. Effect of site, *Acer* species, presence of green islands or leaf status on bacterial and fungal richness, alpha diversity values and comnmunity composition.

	Bacterial endophyte community				Fungal endophyte community		
	Richness	Diversity	Faith’s PD	Composition	Richness	Diversity	Composition
**All samples**							
Site	+	+	+	+	—	—	+
*Acer* species	o	+	o	—	—	—	—
Green islands	—	—	—	—	+	+	—
Leaf status	—	—	—	—	+	+	+
*A. campestre*							
Site	+	—	+	+	—	—	+
Green islands	—	—	—	NA	+	+	NA
Leaf status	—	—	—	—	o	o	+
***A. platanoides***							
Site	+	+	+	+	—	—	+
Green islands	—	—	—	NA	o	—	NA
Leaf status	—	—	—	—	—	—	—

+, significant (≤0.05); − not significant (>0.05); o, marginally significant (≤0.1).

Alpha diversity values are represented by richness (number of observed zOTUs), diversity (Shannon diversity index H’) and phylogenetic diversity (Faith’s PD). Results for community composition are based on model 1 (model with leaf status) and Bray-Curtis dissimilarities.

Abbreviations: NA, not available (not calculated as there was no effect of green island presence on endophyte community composition when testing the entire dataset).

### Bacterial and fungal taxa associated with leaf status and *Acer* species

**Figure 4:**
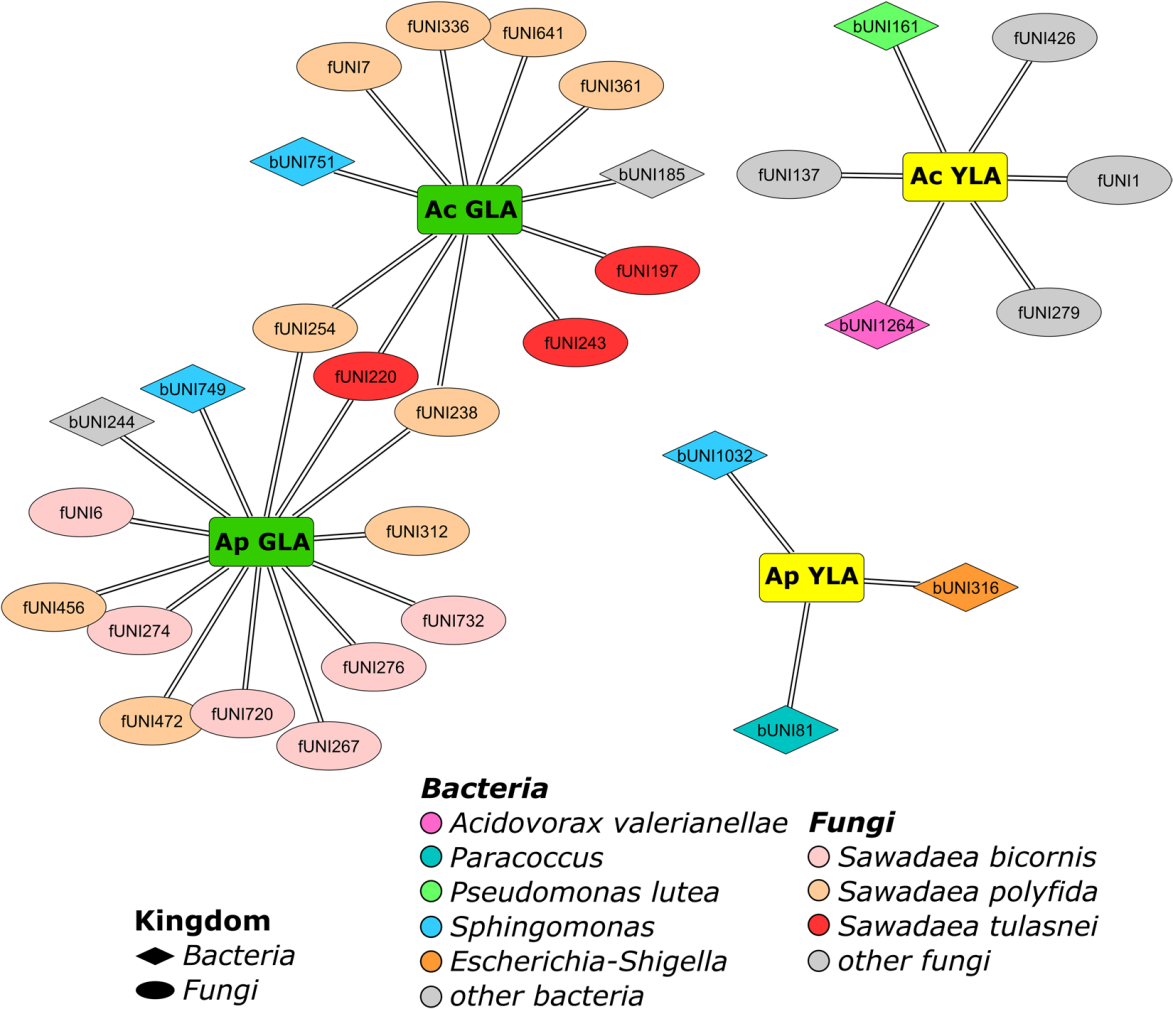
Bacterial and fungal zOTUs significantly associated with yellow (YLA) and green leaf areas (GLA) of *A. campestre* (Ac) and *A. plantanoides* (Ap) leaves with green islands.

To identify bacterial and fungal taxa responsible for the observed differences among leaf status and *Acer* species, we performed an indicator species analysis (Fig. 4, Supplementary Material Table S8). We detected a lower number of significantly associated bacterial (n = 72) than fungal (n = 106) zOTUs. In general, the number of significantly associated fungal and bacterial zOTUs was lower for *A. platanoides* than for *A. campestre*. The highest number of associated bacterial (n = 53) and fungal (n = 48) zOTUs was observed for CL samples of *A. campestre*.

Several zOTUs belonging to the Mycosphaerellaceae were significantly associated with CL samples of both *Acer* species. Four fungal zOTU of this family were significantly affiliated with leaf status YLA of *A. campestre*, while no fungal zOTU was associated with YLA samples of *A. platanoides*.

Six zOTUs belonging to *S. polyfida* and three zOTUs of *S. tulasnei* were significantly linked to leaf status GLA of *A. campestre*. Six zOTUs of *S. bicornis*, five zOTUs of *S. polyfida* and one zOTU belonging to *S. tulasnei* were significantly associated with leaf status GLA of *A. platanoides*. Three of these zOTUs were linked to leaf status GLA in both *Acer* species. In addition, we identified two bacterial zOTUs (*Pseudomonas lutea* and *Acidovorax valerianellae*), which were significantly associated with YLA samples of *A. campestre*. The three bacterial zOTUs assigned to *Paracoccus* sp. K3B-8, *Sphingomonas rubra* and *Escherichia-Shigella* were significantly affiliated with YLA samples of *A. platanoides*. Only two bacterial zOTUs were significantly associated with GLA samples of *A. campestre* (one unidentified *Sphingomonas* and one unidentified bacterium of Acetobacteraceae) and *A. platanoides* (one *Sphingomonas* sp. Leaf339 and one unidentified bacterium belonging to P3OB-42 of the Myxococcales).

## Discussion

Studies on fungal and bacterial endophytes and their interactions in non-healthy plant tissues are still lacking. In the current study, we assessed fungal and bacterial endophyte communities in leaves with and without green islands of *A. campestre* and *A. platanoides*. Consistent with previous research^29,30^, bacterial communities of both *Acer* species were dominated by Gammaproteobacteria. The predominant fungal classes in our study were Dothideomycetes and Leotiomycetes. Our results are only partly in line with previous studies on foliar fungal endophytes of trees^30–33^. We assume that the contrasting findings in endophyte communities observed in our and other studies are related to differences in sampling site and plant species investigated. Previous studies showed that sampling site^9,34–36^ and plant species^4,6,36^ are main drivers of foliar endophyte communities. This is only partly supported by our study. *Acer* tree species had no effect on fungal and bacterial alpha diversity values (except bacteria’s Shannon diversity index). However, we cannot exclude that the differences observed for the bacterial diversity detected between the two *Acer* species are the result of an indirect sampling site effect as we did not collect *A. platanoides* leaves at site Friedland.

Neither leaf status nor the presence of green islands influenced bacterial community composition. In contrast, Ren *et al*.^24^ observed differences in the composition of bacterial communities in needles of asymptomatic and symptomatic (*Heterobasidion*-rotten) *Picea abies* trees. In another study on bacterial endophyte communities in citrus roots, infection by Candidatus *Liberibacter asiaticus* had a strong effect on structure and composition of these communities^37^. This is in line with a study of Koskella *et al*.^38^ who showed that tissue type (asymptomatic vs. symptomatic) significantly affected bacterial community composition in the bark of horse chestnut (*Aesculus hippocastanum*) trees.

Green island presence had no effect on fungal endophyte community composition. Similarly, Abdelfattah *et al*.^27^ found no significant differences in fungal composition in citrus leaves asymptomatic and symptomatic for greasy spot disease. They suggested that the putative pathogen responsible for this disease is present in asymptomatic leaves as latent infections. This conclusion is supported by a recent study on fungal diversity in ash leaves infected by the invasive ascomycete *Hymenoscyphus fraxineus*^39^. Here, symptoms of necrotic leaf lesions were observed after a long latent phase, although a continuous accumulation of pathogen biomass in ash leaves from the initiation of sporulation throughout the season was detected. Previous studies showed that several fungal endophytes are latent pathogens^40,41^ and can switch their life strategy when the host/the plant tissues senesces^42,43^. In the current study, we did not detect other symptoms or signs of fungal infections such as mildew patches. We hypothesize that such symptoms would be more prominent in senescent leaves.

We found significant differences in the fungal community composition between YLA and GLA samples of *A. campestre*. In addition, we observed a lower fungal richness and diversity in GLA samples of *A. campestre* but not of *A. platanoides*. Interestingly, alpha diversity values were not affected by both presence of green islands and leaf status. Previous studies investigating bacterial and fungal endophyte diversity in diseased and apparently healthy plant tissues found similar contrasting results^9,21,23,27^. For instance, no significant differences in fungal richness and diversity between healthy and dieback-diseased plants of five invasive species across Northern Australia were detected by Raghavendra *et al*.^44^. Contrarily to our results for bacterial alpha diversity values, Bogas *et al*.^21^ observed a higher bacterial diversity in *Paullina cupana* plants associated with symptomatic anthracnose (caused by *Colletotrichum* spp.) compared to asymptomatic plants, indicating interactions between anthracnose and the bacterial endophytic communities investigated.

We speculate that interactions between fungal endophytes are responsible for the observed differences in fungal community composition and lower alpha diversity values in GLA samples. As endophytes are supposed to “guard their turf” from potential usurpers (see^43^), fungal species occupying plant tissues should block other species from invading these areas. Yan *et al*.^45^ have elegantly shown that fungal endophytes boost their diverse arsenal of secondary compounds aiming to fence their tissues from invasion by other colonizing species. On the other hand, as endophytic taxa have quite different life history traits resulting in functional complementarity, they may occupy even closely related niches within plant tissues^46^.

Another possible explanation for the observed differences between fungal communities in GLA and YLA samples of *A. campestre* is that the fungal endophytes differ in their niche preferences, as Mycosphaerellaceae and *S. polyfida* dominated YLA and GLA samples, respectively. This supports a previous study of Ernst *et al*.^47^ on fungal endophytes of *Phragmites australis*. The authors observed that three unrelated species of the Ascomycota differed in their niche preferences (i.e., host organ, host habitat, and time of season) and concluded that spatio-temporal niche partitioning allowed for differential colonization of common reed by the fungal species investigated.

The high abundance of powdery mildew genus *Sawadaea* in GLA samples and the results of the indicator species analysis suggests that these fungi could be responsible for the green islands in the *Acer* leaves. Members belonging to this genus are obligate parasitic fungi of several maple species and have a limited host range^48^. However, other symptoms or signs of fungal infections supporting this conclusion were not detected. As a consequence, future studies over longer time periods are needed to identify the cause of the green islands observed in the current study.

Finally, we showed that fungal and bacterial communities responded in different ways towards the factors investigated, supporting the need for synergy between bacterial and fungal endophyte research efforts^2^. Similar different responses of fungal and bacterial endophytes towards abiotic and biotic factors have been observed in two previous studies^5,7^. The observed different responses in our and the above-mentioned studies might be related to differences in the lifestyle strategies of fungi and bacteria, which have been described previously^1^. Another possible explanation for the more pronounced response of fungal endophytes towards the presence of green islands is that the fungal pathogen competes with other fungi for the same ecological niche^49,50^. Negative associations between co-occurrence of fungal endophyte species were observed for *Cirsium arvense* by Gange *et al*.^36^. The authors suggested that these findings are related to the sequence of endophyte colonization. As a consequence, future studies should investigate endophyte communities and their interactions spanning the whole lifetime of leaves (see^39^).

A few technical and biological limitations of this study must be considered. We cannot exclude the possibility that at least part of the bacteria and fungi detected in our study are not actual endophytes but saprotrophs originating from leaf surfaces or the soil, although we collected freshly fallen leaves only. In addition, more leaf samples collected from different sites over a longer sampling period should be analyzed to better understand the causal agents for the green islands and the multiple interactions of endophyte communities. Future studies should also perform epidemiological analyses to support the conclusion that the *Sawadaea* spp. are responsible for the green islands observed in the *Acer* leaves.

## Conclusion

Studies on fungal and bacterial endophytes and their interactions in non-healthy plant tissues are still scarce. The current study provides first insights into the diversity and community composition of fungal and bacterial endophytes in leaves with and without green islands of two maple tree species. The high abundance of *S. polyfida* and *S. bicornis* in GLA samples of *A. campestre* and *A. platanoides*, respectively, suggests that these fungi could be responsible for the green islands in the *Acer* leaves. We cannot, however, exclude that other microorganisms or the host species are responsible for the formation of green islands. Fungal endophyte communities appeared to be more affected by leaf status and/or presence of green islands than bacterial endophytes. However, the effects of green island presence or leaf status on fungal endophytes were *Acer* species-dependent: only fungal endophytes in leaves of *A. campestre* were affected by these factors. As a consequence, more research on endophyte communities in leaves with and without green islands of *Acer* trees collected at different sites over a longer time period as well as competition experiments with *S. polyfida* and *S. bicornis* are needed to unravel the diverse plant-microbiome-pathogen interactions in leaves with green islands.

## Materials and Methods

### Study site and sampling

Leaves with and without green islands (Fig. 1) of at least four *A. platanoides* and *A. campestre* trees per site and *Acer* tree species were collected at two sites in Göttingen (Molkengrund, E 9°57′39″/N 51°32′15″; Am Kehr, E 9°58′46″/N 51°31′36″) at 1^st^ November 2015 (Table 1). Every single leaf was collected directly under a single tree canopy (number of trees per site and *Acer* species: ≥4; Table 1). Only those leaves were sampled, which were clearly fallen from the tree in the last hours (i.e., only those leaves, which lay loosely on top of the litter layer). As there were only few *A. campestre* leaves without green islands, control leaves of approximately ten *A. campestre* trees were also collected in a forest next to Friedland (E 9°56′17″/N 51°25′13″). Each leaf was separately placed in a sterile bag and transported to the laboratory on ice within a few hours. The leaves were stored at −20 °C.

### Surface sterilization of plant material and DNA extraction

Leaves were surface-sterilized by serial washing in 70% ethanol for 2 min, 2% sodium hypochlorite for 3 min and 70% ethanol for 1 min, followed by two times immersion in sterile, distilled water for 45 s and once in sterile, diethylpyrocarbonate (DEPC)-treated water. Surface-sterilized leaves were subsequently dried on sterile tissue paper. Fresh solutions and separate, sterile collection tubes were used for each leaf to avoid cross-contaminations. The effectiveness of the applied sterilization process was controlled as described previously^51^. Briefly, aliquots of the water used in the final wash step were subjected to PCR targeting the bacterial 16S rRNA gene and the fungal ITS2 region. No PCR products were detected. In addition, water from the same aliquots was plated on common laboratory media plates. No growth of fungi or bacteria was observed after incubation in the dark at 25 °C for at least one week. These results confirmed that the surface sterilization of all *Acer* leaves was successful in eliminating cultivable as well as non-cultivable epiphytic fungi and bacteria as well as potential DNA traces from the leaf surfaces.

Five to ten fragments of approximately 10 mm diameter were cut of eight (*A. platanoides*) and nine (*A. campestre*) leaves without green islands (control, CL) using sterile scalpels. In addition, five to ten leaf fragments of around 10 mm diameter were cut from green islands as well as from the surrounding yellow tissues from twelve *A. platanoides* and twelve *A. campestre* leaves. We chose only leaves without any other symptoms of diseases, such as lesions. All fragments of the same leaf were pooled based on their leaf status (CL, YLA and GLA) and stored at −20 °C until DNA extraction. Total DNA of pooled leaf fragments was extracted employing the peqGOLD Plant DNA Mini kit (Peqlab, Erlangen, Germany; now VWR) according to the manufacturer’s instructions with two modifications described previously^52^. In addition, all leaf fragments were incubated in lysis buffer at 32 °C for 12 hours and subsequently homogenized using ethanol-sterilized pestles. Concentration of DNA extracts was quantified using a NanoDrop ND-1000 spectrophotometer (NanoDrop Technologies, Wilmington, USA). In total, DNA of 65 *Acer* leaf samples was subjected to PCR targeting the bacterial 16S rRNA gene and the fungal ITS region (Supplementary Material Table S1).

### Amplification of the 16S rRNA gene

Bacterial endophyte communities in *A. platanoides* and *A. campestre* leaves were assessed by a nested PCR approach targeting the 16S rRNA gene. For details of the first PCR reaction mixture and the thermal cycling scheme see Wemheuer *et al*.^53^. In brief, the primers 799f (5′-AACMGGATTAGATACCCKG-3′)^54^ and 1492R (5′-GCYTACCTTGTTACGACTT-3′)^55^ were used in the first PCR to suppress co-amplification of chloroplast-derived 16S rRNA genes. Bacteria-specific bands were purified and quantified as described in Wemheuer and Wemheuer^51^. Purified PCR products were subjected to nested PCR.

The V6−V8 region of the 16S rRNA gene was amplified with primers 968F and 1401R^56^ containing MiSeq adaptors (underlined) (MiSeq-968F 5′-TCGTCGGCAGCGTCAGATGTGTATAAGAGACAGAACGCGAAGAACCTTAC-3′; MiSeq- 1401R 5′-GTCTCGTGGGCTCGGAGATGTGTATAAGAGACAGCGGTGTGTACAAGACCC-3′) as described previously^51^ with one modification: 0.5 U of Phusion high fidelity DNA polymerase (Thermo Scientific, Waltham, MA, USA) was used. Three independent PCRs were performed per sample. Obtained PCR products were controlled for appropriate size, pooled in equal amounts and purified using the peqGOLD Gel Extraction kit (Peqlab). Quantification of the PCR products was performed using the Quant-iT dsDNA HS assay kit and a Qubit fluorometer (Thermo Scientific) as recommended by the manufacturer. Purified PCR products were barcoded using the Nextera XT-Index kit (Illumina, San Diego, USA) and the Kapa HIFI Hot Start polymerase (Kapa Biosystems, Wilmington, USA). The Göttingen Genomics Laboratory determined the sequences of the partial 16S rRNA genes employing the MiSeq Sequencing platform and the MiSeq Reagent Kit v3 (2 × 300 cycles) as recommended by the manufacturer (Illumina).

### Amplification of the ITS region

Fungal endophyte communities in leaves of *A. platanoides* and *A. campestre* leaves were assessed by a nested PCR approach targeting the ITS region as described previously^51^. In the first PCR, the primers ITS1-F_KYO2 (5′-TAGAGGAAGTAAAAGTCGTAA-3′)^57^ and ITS4 (5′- TCCTCCGCTTATTGATATGC-3′)^58^ were used to suppress co-amplification of plant-derived ITS regions. Obtained PCR products were purified and quantified as described for the bacterial PCR products. The ITS2 region was subsequently amplified using approximately 50 ng product of the first PCR and the primers ITS3_KYO2^57^ and ITS4^58^ containing the MiSeq adaptors (underlined): MiSeq-ITS3_KYO2 (5′-TCGTCGGCAGCGTCAGATGTGTATAAGAGACAGGATGAAGAACGYAGYRAA-3′) and MiSeq-ITS4 (5′-GTCTCGTGGGCTCGGAGATGTGTATAAGAGACAGTCCTCCGCTTATTGATATGC -3′) as described previously^51^. Three independent PCRs were performed per sample. Obtained PCR products were pooled in equal amounts, purified and quantified as described for bacterial PCR products. Purified PCR products were barcoded using the Nextera XT-Index kit (Illumina) and the Kapa HIFI Hot Start polymerase (Kapa Biosystems). The Göttingen Genomics Laboratory determined the sequences of the ITS2 region employing the MiSeq Sequencing platform and the MiSeq Reagent Kit v3 (2 × 300 cycles) as recommended by the manufacturer (Illumina).

### Processing of bacterial and fungal datasets

Obtained sequencing data were initially quality filtered with the Trimmomatic tool version 0.36^52^. Low quality reads were truncated if the quality dropped below 15 in a sliding window of 4 bp. Subsequently, all reads shorter than 100 bp and orphan reads were removed. Remaining sequences were merged, quality-filtered and further processed with USEARCH version 10.0.240^59^. Filtering included the removal of reads shorter than 400 bp or longer than 470 bp (bacteria) or shorter than 220 bp and longer than 460 bp (fungi) as well as the removal of low-quality reads (expected error >1) and reads with more than one ambiguous base.

Processed sequences of all samples were combined, and redundant sequences were subsequently removed. Obtained unique sequences were denoised and clustered in zOTUs (i.e., sequences with 100% similarity) with the unoise3 algorithm^60^ implemented in USEARCH^59^. Chimeric sequences were removed *denovo* using the UCHIME algorithm during clustering^61^. Subsequently, remaining chimeric sequences were removed using UCHIME^62^ in reference mode with the SILVA SSU Ref NR 99 132 database^63^ as reference data set for bacteria and the QIIME release of the UNITE database version 7.2^64^ for fungi. To assign taxonomy of bacteria and fungi, unique and chimera-free sequences were classified by BLAST alignment^65^ against the SILVA^63^ and the UNITE^64^ database, repectively. Taxonomy was assigned by stand-alone BLAST using the best-hit approach with an e-value cutoff of 1e-20. The taxonomy of the best hit identified during BLAST search is affiliated to the respective zOTU. Combined sequences of all samples were mapped on the final set of unique sequences to calculate the evenness and abundance of each zOTU in all samples. All non-bacterial or non-fungal zOTUs were removed based on their taxonomic classification in the respective database. In addition, all zOTUs consisting of one single sequence (singletons) were removed prior to statistical analysis. Final zOTUs tables for bacteria and fungi are provided as Supplementary Material Tables S2, S3, respectively. Sequence characteristics for bacterial and fungal dataset are provided as Supplementary Material Tables S4, S5, respectively.

### Data analysis

All data analyses were conducted in R version 3.5.0^66^ or the PRIMER 6.0 software with the PERMANOVA + add-on (PRIMER-E, Plymouth Marine Laboratory, UK). Differences were considered as statistically significant with P ≤ 0.05 and as marginally significant with P ≤ 0.1. Bacterial and fungal communities were analysed separately. Alpha diversity indices (richness, Shannon index of diversity, Faith’s phylogenetic diversity, Chao1 and Michaelis-Menten Fit) were calculated using the R-packages *vegan* 2.5-2^67^, *picante* version 1.7^61^ and *drc* version 3.0-1^68^. The zOTU tables were rarefied to 8,061 (bacteria) or 11,932 (fungi) sequences per sample prior to alpha diversity analysis using the *rrarefy* function in *vegan*^67^.

Diversity was calculated using *diversity* function in *vegan*^67^. Sample coverage was estimated using the Michaelis-Menten Fit calculated in R. For this purpose, richness and rarefaction curves were calculated using the *specnumber* and the *rarecurve* function, respectively, in the *picante* package^61^. The Michaelis-Menten Fit was subsequently calculated from generated rarefaction curves using the *MM2* model within the *drc* package^68^. The alignment and the phylogenetic tree necessary for the calculation of bacterial Faith’s phylogenetic diversity were constructed in MUSCLE v3.8.425^69^ using the neighbour-joining algorithm. All alpha diversity indices were calculated 10 times. The average of all iterations was used for further statistical analyses. Final tables containing bacterial and fungal alpha diversity values are provided as Supplementary Material Tables S4, S5, respectively.

Alpha diversity data were tested for normal distribution with *shapiro* function and for homogeneity of variance with *leveneTest* function using the R package *car* version 3.0-3^70^. Because the distribution of microbial diversity, phylogenetic diversity and richness significantly differed from a normal distribution, changes in alpha diversity values were evaluated by linear mixed effects models using the R packages *lmerTest* version 3.0-1^71^ and *MuMIn* version 1.42.1^72^. As fixed effects, we entered *Acer* species and leaf status or presence of green islands. We also tested for significant interactions between site and *Acer* species and between leaf status and *Acer* species. The random factor considered in each model was site. The best model was chosen according to lowest Akaike information criterion (AIC). The sample size was relatively small in comparison to the number of estimated parameters. As a consequence, AICc was used for model selection in the R package *MuMIn*^72^. Visual inspection of residual plots did not reveal any obvious deviations from homoscedasticity or normality.

The final model was calculated using the function *lmer* provided within *lmerTest* with restricted maximum likelihood. P-values were obtained by likelihood ratio tests of the full model with the effect in question against the model without the effect in question. Significance levels for fixed factors and their interaction are based on F-values, calculated by a type III analysis of variance with Satterthwaite approximation for degrees of freedom within the R package *lmerTest*^71^. Statistically and marginally significant results of fixed factors were followed up with Dunn’s test for multiple comparisons with *Benjamini-Hochberg* correction using the R-package FSA^73^. For the random factor site, an ANOVA-like table with likelihood ratio test statistics was generated using the *ranova* function in the R package *lmerTest*^71^.

Overall patterns of the entire bacterial and fungal community structure were analysed by permutational multivariate analysis of variance (PERMANOVA; Typ III) with 999 random permutations using the PRIMER 6.0 software with the PERMANOVA + add-on (PRIMER-E, Plymouth Marine Laboratory, UK). The same factors as described for alpha diversity analysis were included in the design. Four different dissimilarity measures were tested for the bacterial dataset: Bray-Curtis, Jaccard, and weighted as well as unweighted UniFrac. For the fungal dataset, only Bray-Curtis and Jaccard were tested due to the high variability of the ITS region which is inappropriate for phylogenetic analyses. The matrices were calculated in R using the *vegdist* and *GUniFrac* functions within the R packages *vegan* and *GUniFrac*^74^, respectively. The same bacterial tree used for Faith’s phylogenetic diversity was used for the calculation of UniFrac distances. Pre-analyses revealed that Bray-Curtis and/or weighted UniFrac dissimilarities displayed a higher environmental sensitivity based on the higher coefficients of determination (data not shown). As a consequence, only results for these distance measures are shown here. Differences in community structure between the three leaf states (CL, YLA and GLA) were tested using the PRIMER 6.0 software. Differences in community structure were visualized using the *metaMDS* function in the *vegan* package^67^.

To identify potential indicators as well as highly associated zOTUs for each *Acer* species with regard to leaf status, multipattern analyses were applied. For this purpose, the *multipatt* function from the *indicspecies* package^28^ was used. The biserial coefficients (*R*) with a particular leaf status or *Acer* species were corrected for unequal sample size using the function *r.g*^75^. The analysis was performed for each *Acer* species individually (Supplementary Material Table S8). To enhance reliability of the indicator analysis, only fungal and bacterial zOTUs found in at least five samples of either *A. campestre* or *A. plantanoides* were considered. Associated fungal and bacterial zOTUS of leaf states GLA and YLA were visualized using *Cytoscape* version 3.6.1^76^.

## Supplementary information

Supplementary Information

Table S1

Table S2

Table S3

Table S4

Table S5

Table S6

Table S7

Table S8

## Data Availability

Sequence data were deposited in the sequence read archive (SRA) of the National Center for Biotechnology Information (NCBI) under accession number SUB4399606 (bacteria) and SUB4399627 (fungi).

## References

[1] Hardoim PR (2015). The hidden world within plants: ecological and evolutionary considerations for defining functioning of microbial endophytes. Microbiol. Mol. Biol. Rev..

[2] Le Cocq K, Gurr SJ, Hirsch PR, Mauchline TH (2016). Exploitation of endophytes for sustainable agricultural intensification. Mol. Plant Pathol..

[3] van Overbeek LS, Saikkonen K (2016). Impact of Bacterial–Fungal Interactions on the Colonization of the Endosphere. Trends Plant Sci..

[4] Wemheuer F (2017). Bacterial endophyte communities of three agricultural important grass species differ in their response towards management regimes. Sci. Rep..

[5] Granzow, S. *et al*. The effects of cropping regimes on fungal and bacterial communities of wheat and faba bean in a greenhouse pot experiment differ between plant species and compartment. *Front. Microbiol*. **8**, 10.3389/fmicb.2017.00902 (2017). 10.3389/fmicb.2017.00902 PMC5447230 28611735

[6] Shen, S. Y. & Fulthorpe, R. Seasonal variation of bacterial endophytes in urban trees. *Front. Microbiol*. **6**, 10.3389/fmicb.2015.00427 (2015). 10.3389/fmicb.2015.00427 PMC4437045 26042095

[7] Coleman-Derr D (2016). Plant compartment and biogeography affect microbiome composition in cultivated and native *Agave* species. New Phytol..

[8] Broberg M, Doonan J, Mundt F, Denman S, McDonald JE (2018). Integrated multi-omic analysis of host-microbiota interactions in acute oak decline. Microbiome.

[9] Blaustein RA, Lorca GL, Meyer JL, Gonzalez CF, Teplitski M (2017). Defining the core *Citrus* leaf- and root-associated microbiota: Factors associated with community structure and implications for managing Huanglongbing (*Citrus* greening) disease. Appl. Environ. Microb..

[10] Kemen E (2014). Microbe–microbe interactions determine oomycete and fungal host colonization. Curr. Opin. Plant Biol..

[11] Walters DR, McRoberts N, Fitt BD (2008). Are green islands red herrings? Significance of green islands in plant interactions with pathogens and pests. Biol. Rev. Camb. Philos. Soc..

[12] Jameson PE (2000). Cytokinins and auxins in plant-pathogen interactions – An overview. Plant Growth Regul..

[13] Scott KJ (1972). Obligate parasitism by phytopathogenic fungi. Biol. Rev..

[14] Glawe DA (2008). The powdery mildews: a review of the world’s most familiar (yet poorly known) plant pathogens. Annu. Rev. Phytopathol..

[15] Smart CM, Scofield SR, Bevan MW, Dyer TA (1991). Delayed leaf senescence in tobacco plants transformed with *tmr*, a gene for cytokinin production in *Agrobacterium*. Plant Cell.

[16] Walters DR, McRoberts N (2006). Plants and biotrophs: a pivotal role for cytokinins?. Trends Plant Sci..

[17] Engelbrecht L, Orban U, Heese W (1969). Leaf-miner caterpillars and cytokinins in the “green islands” of autumn leaves. Nature.

[18] Giron D, Kaiser W, Imbault N, Casas J (2007). Cytokinin-mediated leaf manipulation by a leafminer caterpillar. Biol. Lett..

[19] Kaiser W, Huguet E, Casas J, Commin C, Giron D (2010). Plant green-island phenotype induced by leaf-miners is mediated by bacterial symbionts. Proc Biol Sci.

[20] Tian B-Y, Cao Y, Zhang K-Q (2015). Metagenomic insights into communities, functions of endophytes, and their associates with infection by root-knot nematode, *Meloidogyne incognita*, in tomato roots. Sci. Rep..

[21] Bogas AC (2015). Endophytic bacterial diversity in the phyllosphere of Amazon *Paullinia cupana* associated with asymptomatic and symptomatic anthracnose. SpringerPlus.

[22] Bruez, E. *et al*. Bacteria in a wood fungal disease: characterization of bacterial communities in wood tissues of esca-foliar symptomatic and asymptomatic grapevines. *Front. Microbiol*. **6**, 10.3389/fmicb.2015.01137 (2015). 10.3389/fmicb.2015.01137 PMC4621878 26579076

[23] Douanla-Meli C, Langer E, Talontsi Mouafo F (2013). Fungal endophyte diversity and community patterns in healthy and yellowing leaves of *Citrus limon*. Fungal Ecol..

[24] Ren Fei, Kovalchuk Andriy, Mukrimin Mukrimin, Liu Mengxia, Zeng Zhen, Ghimire Rajendra P., Kivimäenpää Minna, Holopainen Jarmo K., Sun Hui, Asiegbu Fred O. (2018). Tissue Microbiome of Norway Spruce Affected by Heterobasidion-Induced Wood Decay. Microbial Ecology.

[25] de Assis Costa OY, Tupinambá DD, Bergmann JC, Barreto CC, Quirino BF (2018). Fungal diversity in oil palm leaves showing symptoms of Fatal Yellowing disease. PLOS ONE.

[26] Deveau A (2018). Bacterial–fungal interactions: ecology, mechanisms and challenges. FEMS Microbiol. Rev..

[27] Abdelfattah A, Cacciola SO, Mosca S, Zappia R, Schena L (2017). Analysis of the fungal diversity in *Citrus* leaves with greasy spot disease symptoms. Microb. Ecol..

[28] De Cáceres M, Legendre P (2009). Associations between species and groups of sites: indices and statistical inference. Ecology..

[29] Yang R, Liu P, Ye W (2017). Illumina-based analysis of endophytic bacterial diversity of tree peony (*Paeonia* Sect. Moutan) roots and leaves. Braz. J. Microbiol..

[30] Jakuschkin B (2016). Deciphering the pathobiome: intra- and interkingdom interactions involving the pathogen *Erysiphe alphitoides*. Microb. Ecol..

[31] Yang, T., Sun, H., Shen, C. & Chu, H. Fungal assemblages in different habitats in an Erman’s birch forest. *Front. Microbiol*. **7**, 10.3389/fmicb.2016.01368 (2016). 10.3389/fmicb.2016.01368 PMC5003828 27625646

[32] Kemler M (2013). Ion Torrent PGM as tool for fungal community analysis: a case study of endophytes in *Eucalyptus grandis* reveals high taxonomic diversity. PLOS ONE.

[33] Oono R, Lefèvre E, Simha A, Lutzoni F (2015). A comparison of the community diversity of foliar fungal endophytes between seedling and adult loblolly pines (*Pinus taeda*). Fungal Biol..

[34] Proença DN (2017). The microbiome of endophytic, wood colonizing bacteria from pine prees as affected by pine wilt disease. Sci. Rep..

[35] Vega FE (2010). Fungal endophyte diversity in coffee plants from Colombia, Hawai’i, Mexico and Puerto Rico. Fungal Ecol..

[36] Gange AC, Soma D, Amanda FC, Sutton BC (2007). Site- and species-specific differences in endophyte occurrence in two herbaceous plants. J. Ecol..

[37] Trivedi P, Duan Y, Wang N (2010). Huanglongbing, a systemic disease, restructures the bacterial community associated with *Citrus* roots. Appl. Environ. Microb..

[38] Koskella B, Meaden S, Crowther WJ, Leimu R, Metcalf CJE (2017). A signature of tree health? Shifts in the microbiome and the ecological drivers of horse chestnut bleeding canker disease. New Phytol..

[39] Cross H (2017). Fungal diversity and seasonal succession in ash leaves infected by the invasive ascomycete *Hymenoscyphus fraxineus*. New Phytol..

[40] Porras-Alfaro A, Bayman P (2011). Hidden Fungi, Emergent Properties: Endophytes and Microbiomes. Ann. Rev. Phytopathol..

[41] Photita W, Lumyong S, Lumyong P, McKenzie EHC, Hyde KD (2004). Are some endophytes of *Musa acuminata* latent pathogens?. Fungal Divers..

[42] Promputtha I (2007). A phylogenetic evaluation of whether endophytes become saprotrophs at host senescence. Microb. Ecol..

[43] Herre EA (2005). Tropical plants as chimera: some implications of foliar endophytic fungi for the study of host plant defense, physiology, and genetics. Biotic interactions in the tropics.

[44] Raghavendra AK (2017). Characterisation of above-ground endophytic and soil fungal communities associated with dieback-affected and healthy plants in five exotic invasive species. Fungal Ecol..

[45] Yan JF, Broughton SJ, Yang SL, Gange AC (2015). Do endophytic fungi grow through their hosts systemically?. Fungal Ecol..

[46] Kia SH (2017). Influence of phylogenetic conservatism and trait convergence on the interactions between fungal root endophytes and plants. ISME J..

[47] Ernst M, Neubert K, Mendgen KW, Wirsel SGR (2011). Niche differentiation of two sympatric species of *Microdochium* colonizing the roots of common reed. BMC Microbiol..

[48] Hirose S (2005). Molecular phylogeny and evolution of the maple powdery mildew (*Sawadaea*, Erysiphaceae) inferred from nuclear rDNA sequences. Mycol. Res..

[49] Schlegel M, Dubach V, von Buol L, Sieber TN (2016). Effects of endophytic fungi on the ash dieback pathogen. FEMS Microbiol. Ecol..

[50] Blumenstein K (2015). Nutritional niche overlap potentiates the use of endophytes in biocontrol of a tree disease. BioControl.

[51] Wemheuer, B. & Wemheuer, F. Assessing bacterial and fungal diversity in the plant endosphere in *Metagenomics:* Methods *and Protocols* (eds Streit, W. R. & Daniel, R.) 75–84 (Springer New York, 2017). 10.1007/978-1-4939-6691-2_6 27900685

[52] Bolger AM, Lohse M, Usadel B (2014). Trimmomatic: A flexible trimmer for Illumina sequence data. Bioinformatics.

[53] Wemheuer F (2016). Impact of grassland management regimes on bacterial endophyte diversity differs with grass species. Lett. Appl. Microbiol..

[54] Chelius MK, Triplett EW (2001). The diversity of archaea and bacteria in association with the roots of *Zea mays* L. Microb. Ecol..

[55] Lane, D. J. 16S/23S rRNA sequencing in *Nucleic acid techniques in bacterial systematics* (eds Stackebrandt, E. & Goodfellow, M.) 115–175 (John Wiley & Sons, 1991).

[56] Nübel U (1996). Sequence heterogeneities of genes encoding 16S rRNAs in *Paenibacillus polymyxa* detected by temperature gradient gel electrophoresis. J. Bacteriol..

[57] Toju H, Tanabe AS, Yamamoto S, Sato H (2012). High-Coverage ITS Primers for the DNA-based identification of *Ascomycetes* and *Basidiomycetes* in Environmental Samples. PLoS ONE.

[58] White, T. J., Bruns, T., Lee, S. & Taylor, J. Amplification and direct sequencing of fungal ribosomal RNA genes for phylogenetics In *PCR protocols: a guide to methods and applications***(eds** Innis, M. A., Gelfand, D. H., Sninsky, J. J., & White, T. J.) 315–322 (Academic Press, Inc., New York. 1990).

[59] Edgar RC (2010). Search and clustering orders of magnitude faster than BLAST. Bioinformatics..

[60] Edgar, R. C. Updating the 97% identity threshold for 16S ribosomal RNA OTUs. Vol. 34. *Bioinformatics*. **34**, 2371–2375, 10.1093/bioinformatics/bty113 (2018). 10.1093/bioinformatics/bty113 29506021

[61] Kembel SW (2010). Picante: R tools for integrating phylogenies and ecology. Bioinformatics.

[62] Edgar RC, Haas BJ, Clemente JC, Quince C, Knight R (2011). UCHIME improves sensitivity and speed of chimera detection. Bioinformatics.

[63] Quast C (2013). The SILVA ribosomal RNA gene database project: improved data processing and web-based tools. Nucleic Acids Res..

[64] Tedersoo L (2018). High-level classification of the Fungi and a tool for evolutionary ecological analyses. Fungal Divers.

[65] Camacho C (2009). BLAST+: architecture and applications. BMC Bioinformatics..

[66] R Development Core Team. *R: A Language and Environment for Statistical Computing*. R Foundation for Statistical Computing, Vienna. Available online at: http://www.R-project.org/ (2018).

[67] Oksanen, J. *et al*. Vegan: Community ecology package. R package version 2.4-4 Available online at: https://cran.r-project.org/web/packages/vegan/index.html (2017).

[68] Ritz C, Baty F, Streibig JC, Gerhard D (2016). Dose-response analysis using R. PLOS ONE.

[69] Edgar R. C. (2004). MUSCLE: multiple sequence alignment with high accuracy and high throughput. Nucleic Acids Research.

[70] Fox, J. & Weisberg, S. An R Companion to Applied Regression. Second Edition (Thousand Oaks CA: Sage, 2011).

[71] Kuznetsova, A., Brockhoff, P. B., Christensen, R. H. B. lmerTest Package: Tests in Linear Mixed Effects Models. *J. Stat. Softw*. **82**, 10.18637/jss.v082.i13 (2017).

[72] Barton, K. MuMIn: Multi-Model Inference. R package version 1.42.1. Avaibalbe online at: https://CRAN.R-project.org/package=MuMIn (2018).

[73] Ogle, D. H. FSA: Fisheries Stock Analysis. R package version 0.8.20. (2018).

[74] Chen J (2012). Associating microbiome composition with environmental covariates using generalized UniFrac distances. Bioinformatics.

[75] Tichy L, Chytry M (2006). Statistical determination of diagnostic species for site groups of unequal size. J. Veg. Sci..

[76] Shannon P (2003). Cytoscape: a software environment for integrated models of biomolecular interaction networks. Genome Res..

