# Supplementary material for: Deciphering bacterial and fungal endophyte communities in leaves of two maple trees with green islands
Source: Sci Rep. 2019 Oct 2;9:14183. doi: 10.1038/s41598-019-50540-2 (PMC6775154; doi:10.1038/s41598-019-50540-2)
Supplement: Supplementary file 1 — Supplementary Information [file 41598_2019_50540_MOESM1_ESM.docx]

*For submission to Scientific Reports*

Supplementary Information

**Deciphering bacterial and fungal endophyte communities in leaves of two maple trees with green islands**

Franziska Wemheuer, Bernd Wemheuer, Rolf Daniel, Stefan Vidal


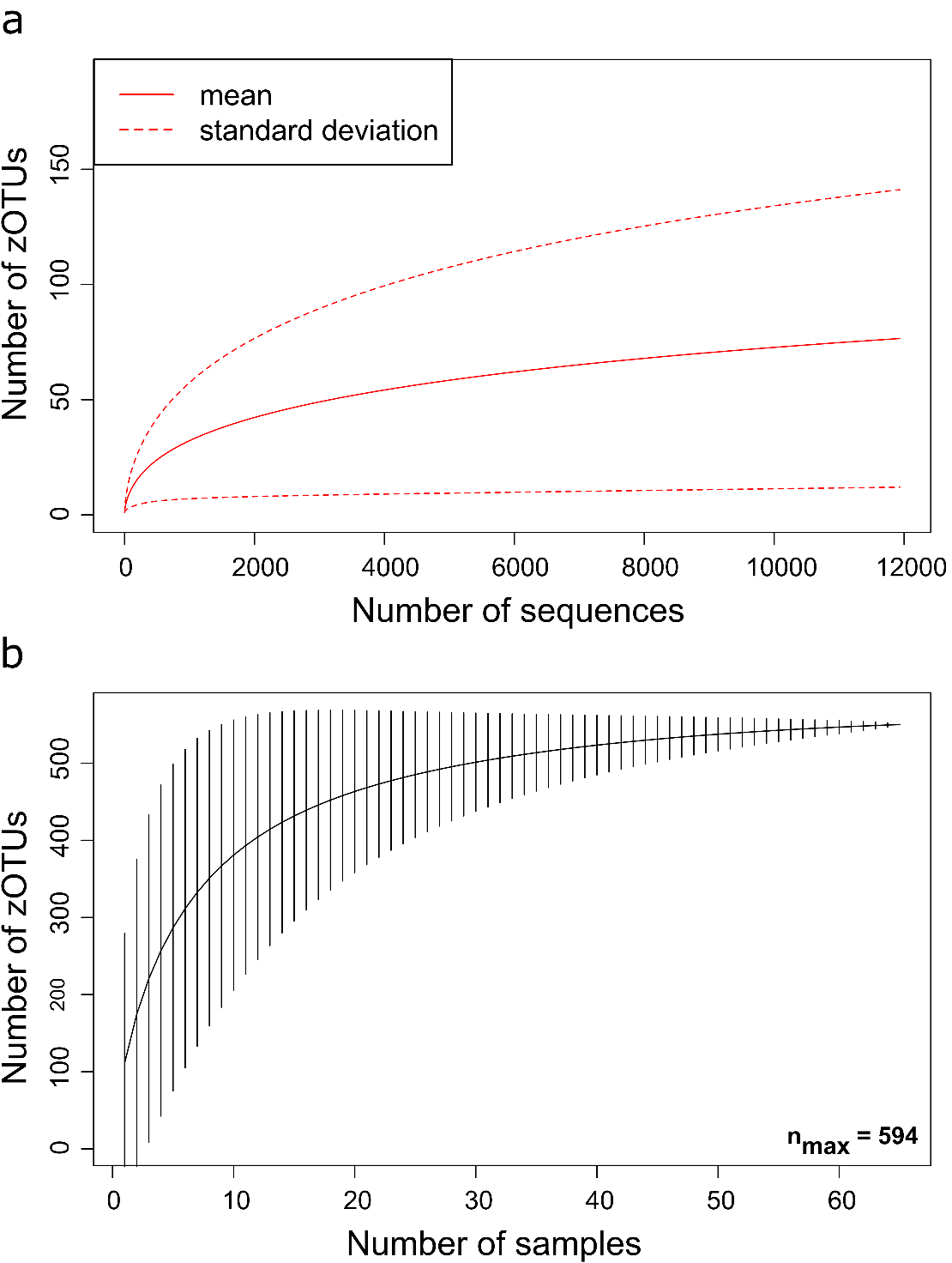


**Figure S1.** Rarefaction (a) and species accumulation (b) curves calculated for the fungal endophyte community in the two investigated *Acer* tree species. Only the mean of all rarefaction curves and the standard deviation are shown.


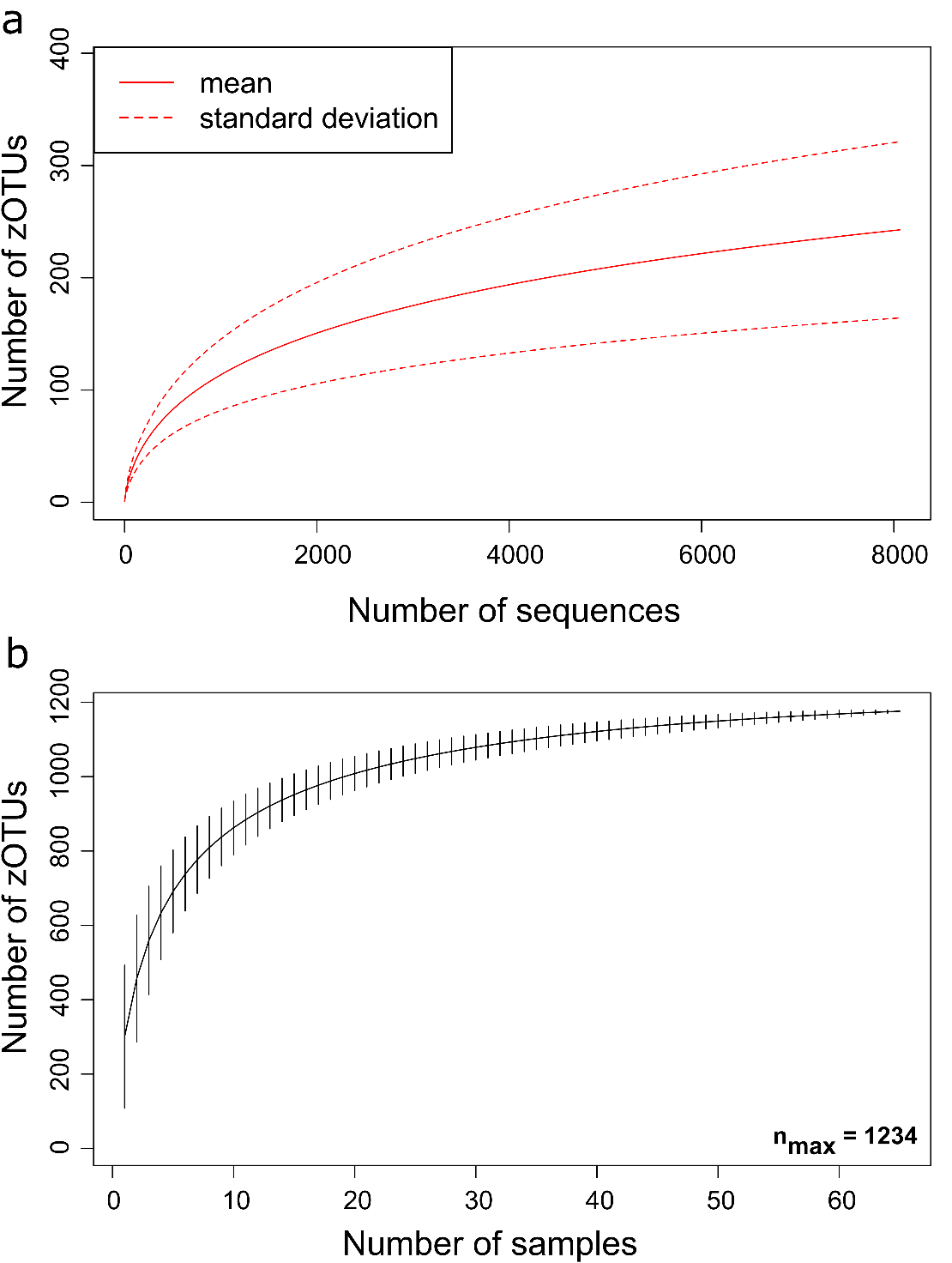


**Figure S2.** Rarefaction (a) and species accumulation (b) curves calculated for the bacterial endophyte community in the two investigated *Acer* tree species. Only the mean of all rarefaction curves and the standard deviation are shown.


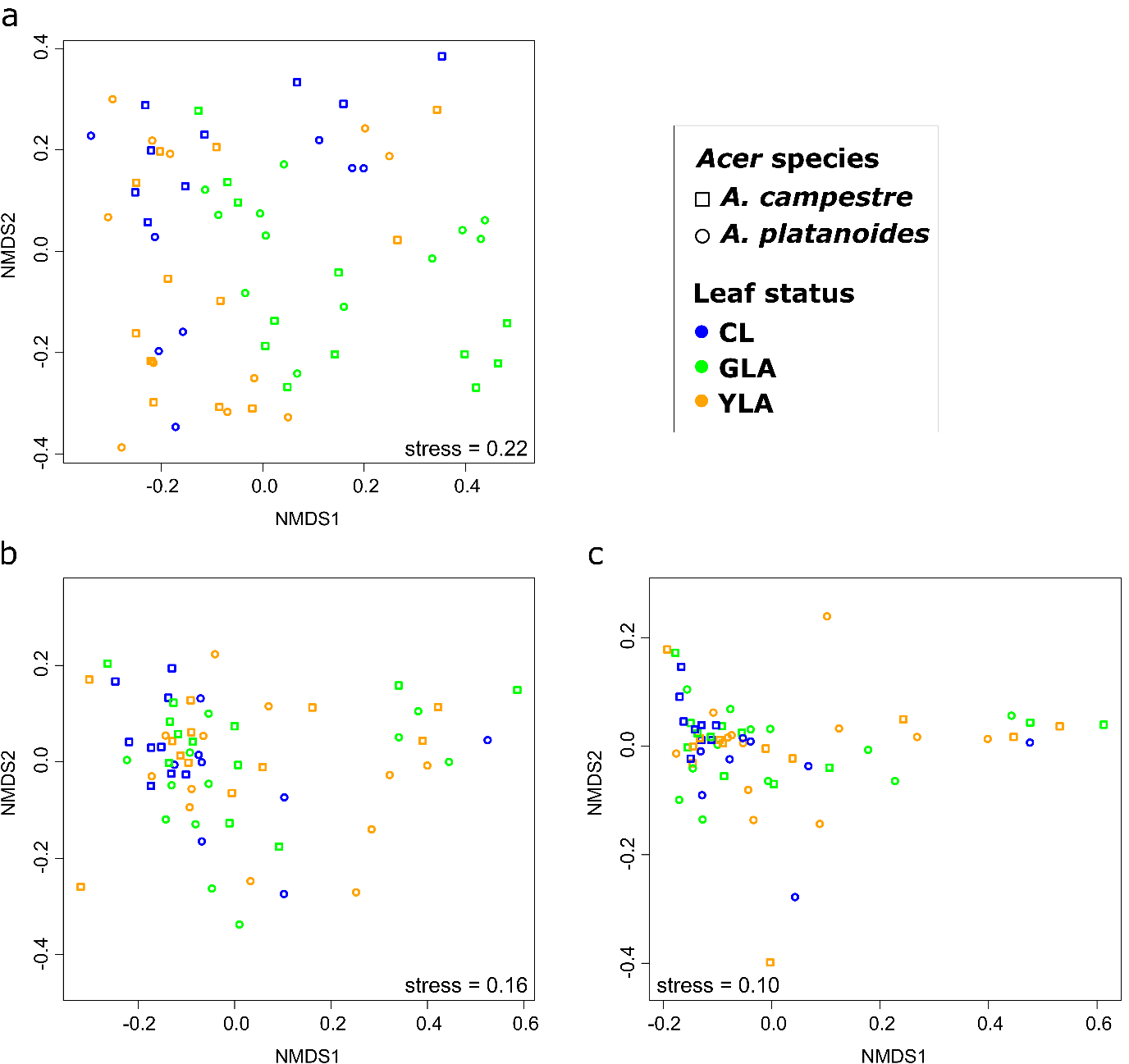


**Figure S3.** Response of fungal (a) and bacterial (b, c) endophyte communities in leaves of *A. campestre* and *A. platanoides* towards leaf status. Ordination is based on Bray-Curtis dissimilarities (a, b) or weighted UniFrac (d) dissimilarities between samples. NMDS ordination of bacterial and fungal communities is color-coded by leaf status. Note that the nMDS axes have different scales for each ordination. Abbreviations: CL, control leaves; YLA, yellow leaf area; GLA, green island area.


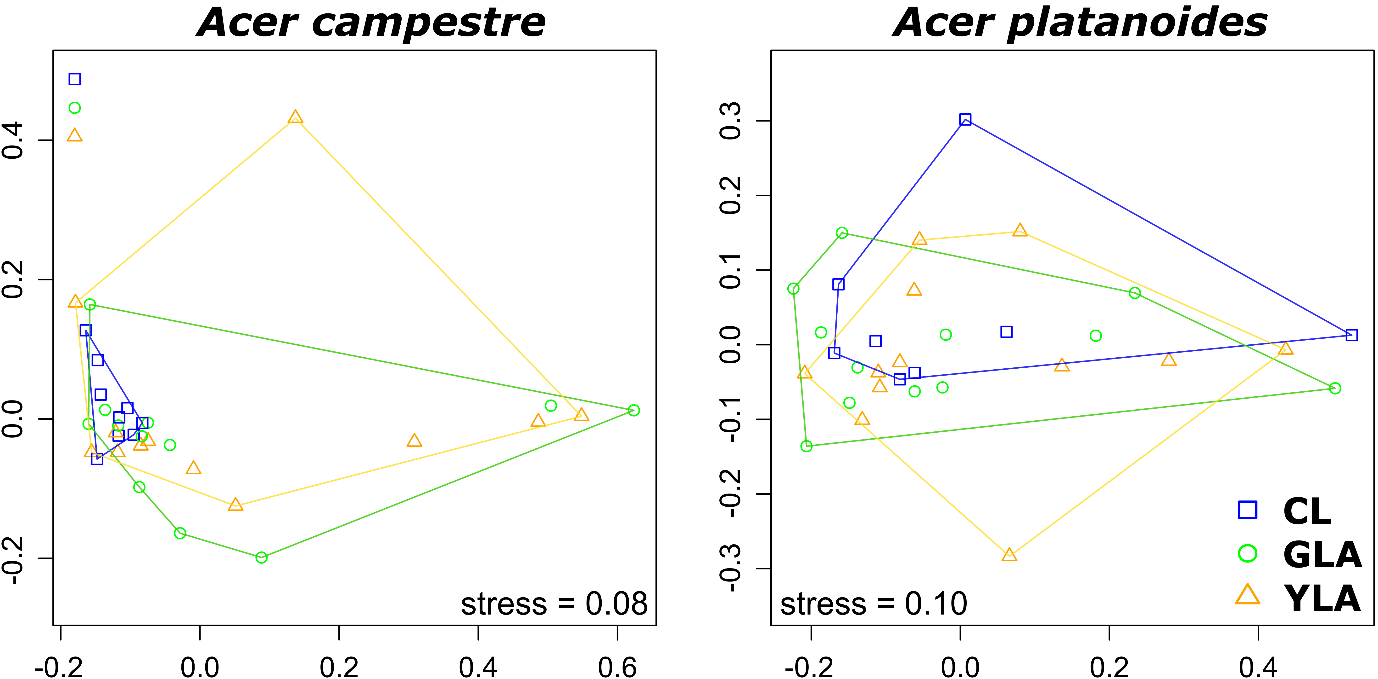


**Figure S4. Response of bacterial endophyte communities in leaves of *A. campestre* and *A. platanoides* towards leaf status**. Ordination is based on weighted UniFrac dissimilarities between samples. NMDS ordination of bacterial and fungal communities is color-coded by leaf status. Note that the nMDS axes have different scales for each ordination. Abbreviations: CL, control leaves; YLA, yellow leaf area; GLA, green island area.

(Supplementary Tables are provided as extra files)

**Table S1. Sample characteristics.**

**Table S2.** **OTU table for the bacterial endophyte community**. For further information on the Sample ID see Table S1 in the Supplementary Material.

**Table S3.** **OTU table for the fungal endophyte community.** For further information on the Sample ID see Table S1 in the Supplementary Material.

**Table S4**. Sequence characteristics (number of sequences) and alpha diversity values of the bacterial dataset. Alpha diversity values were calculated at the same surveying effort (number of sequences = 8,061). Every value was calculated 10 times in R. The average of all 10 iterations is provided.

**Table S5.** **Sequence characteristics (number of sequences) and alpha diversity values of the fungal dataset**. Alpha diversity values were calculated at the same surveying effort (number of sequences = 11,932). Every value was calculated 10 times in R. The average of all 10 iterations is provided.

**Table S6.** **Linear mixed effect model results for alpha diversity values.** Data were analysed with sampling site as random effect and *Acer* species as well as leaf status (CL, YLA, GLA; model 1) or presence of green islands (model 2) as fixed factors. Each *Acer* species was also tested separately (models 3-6). Significance levels for fixed factors are based on F-values, calculated by a type III analysis of variance with Satterthwaite approximation for degrees of freedom. For random factors, an ANOVA-like table with likelihood ratio test (LRT) statistics was generated. Number of observations: 65; number of sites: 3 (entire dataset and *A. campestre*) or 2 (*A. platanoides*), number of leaf states: 3, infection levels: 2, number of *Acer* species: 2. Statistically (P ≤ 0.05) and marginally (P ≤ 0.1) significant results of fixed factors were followed up with Dunn’s test for multiple comparisons with *Benjamini-Hochberg* correction.

**Table S7.** **Results of the PERMANOVA analysis for bacterial endophytes.** Data were analysed with sampling site as random effect and *Acer* species as well as leaf status (model 1) or presence of green islands (model 2) as fixed factors. Each *Acer* species was also tested separately, with site as random and leaf status as fixed factor (models 3 and 4). Results of the PERMANOVA are based on weighted UniFrac dissimilarities with 999 permutations (perm).

**Table S8.** Significantly (P ≤ 0.05) associated bacterial (bUNI) and fungal (fUNI) zOTUs with leaf status*.*
